# Joint analysis of genome-wide genetic variants associated with gene expression and disease susceptibility

**DOI:** 10.1186/gb-2011-12-s1-p29

**Published:** 2011-09-19

**Authors:** Chen-Hsin Yu, John Moult

**Affiliations:** 1 Institute for Bioscience and Biotechnology Research, University of Maryland, Rockville, MD, USA; 2 Molecular and Cellular Biology Program, University of Maryland, College Park, MD, USA; 3 Department of Cell Biology and Molecular Genetics, University of Maryland, College Park, MD, USA

## Background

Genome-wide association studies (GWAS) of human complex disease have identified a large number of disease-associated genetic loci, which are distinguished by distinctive frequencies of specific single nucleotide polymorphisms (SNPs) in individuals with a particular disease. However, these data do not provide direct information on the biological basis of a disease or on the underlying mechanisms. Many studies have shown that variations in gene expression among individuals, as well as among cell types, contribute to phenotype diversity and disease susceptibility. Recent genome-wide expression quantitative trait loci (eQTL) association (GWEA) studies have provided information on genetic factors, especially SNPs, that are associated with gene expression variation. These expression-associated SNPs (exSNPs) have already been utilized to explain some results of GWAS for diseases, but interpretation of the data is handicapped by low reproducibility of the genotype-expression relationships.

## Methods

To address this problem, we established several gold standard sets of high-reliability exSNPs based on multiple occurrences in different GWEA studies in various human populations and cell types. We then related these data to results from GWAS for diseases, to find a set of disease-associated loci that are likely to have an underlying expression mechanism. HapMap linkage disequilibrium data were utilized to allow the comparison of GWEA results from studies that employed different microarray SNP sets.

## Results

We integrated the current gold standard data with SNPs in disease-associated loci from the Wellcome Trust Case-Control Consortium (WTCCC) GWAS of seven common human diseases. Approximately one-third of these disease-associated loci in the WTCCC GWAS were found to be consistent with an underlying expression change mechanism. Comparing separate gold standard sets for Caucasian (CEU), African (YRI) and Asian (ASN) populations also allowed us to investigate which exSNPs contribute to population-specific eQTLs.

## Conclusions

Use of the gold standard set of SNP-expression relationships has enabled us to more reliably determine the role of expression changes in common human diseases.

